# Evaluation of Preprocessing Methods on Independent Medical Hyperspectral Databases to Improve Analysis

**DOI:** 10.3390/s22228917

**Published:** 2022-11-18

**Authors:** Beatriz Martinez-Vega, Mariia Tkachenko, Marianne Matkabi, Samuel Ortega, Himar Fabelo, Francisco Balea-Fernandez, Marco La Salvia, Emanuele Torti, Francesco Leporati, Gustavo M. Callico, Claire Chalopin

**Affiliations:** 1 Research Institute for Applied Microelectronics (IUMA), University of Las Palmas de Gran Canaria, 35017 Las Palmas de Gran Canaria, Spain; 2 Innovation Center Computer-Assisted Surgery (ICCAS), University of Leipzig, 04103 Leipzig, Germany; 3 Center for Scalable Data Analytics and Artificial Intelligence (ScaDS.AI), University of Leipzig, 04105 Leipzig, Germany; 4 Department of Electrical Engineering, Mechanical Engineering and Industrial Engineering, Anhalt University of Applied Science Anhalt, 06366 Köthen, Germany; 5 Nofima, Norwegian Institute of Food Fisheries and Aquaculture Research, NO-9291 Tromsø, Norway; 6 Fundacion Canaria Instituto de Investigación Sanitaria de Canarias (FIISC), 35019 Las Palmas de Gran Canaria, Spain; 7 Department of Psychology, Sociology and Social Work, University of Las Palmas de Gran Canaria, 35017 Las Palmas de Gran Canaria, Spain; 8 Department of Electrical, Computer and Biomedical Engineering, University of Pavia, I-27100 Pavia, Italy

**Keywords:** min-max scaling, standard normal variate normalization, median filter, hyperspectral imaging, machine learning, deep learning, colon cancer, esophagogastric cancer, brain cancer

## Abstract

Currently, one of the most common causes of death worldwide is cancer. The development of innovative methods to support the early and accurate detection of cancers is required to increase the recovery rate of patients. Several studies have shown that medical Hyperspectral Imaging (HSI) combined with artificial intelligence algorithms is a powerful tool for cancer detection. Various preprocessing methods are commonly applied to hyperspectral data to improve the performance of the algorithms. However, there is currently no standard for these methods, and no studies have compared them so far in the medical field. In this work, we evaluated different combinations of preprocessing steps, including spatial and spectral smoothing, Min-Max scaling, Standard Normal Variate normalization, and a median spatial smoothing technique, with the goal of improving tumor detection in three different HSI databases concerning colorectal, esophagogastric, and brain cancers. Two machine learning and deep learning models were used to perform the pixel-wise classification. The results showed that the choice of preprocessing method affects the performance of tumor identification. The method that showed slightly better results with respect to identifing colorectal tumors was Median Filter preprocessing (0.94 of area under the curve). On the other hand, esophagogastric and brain tumors were more accurately identified using Min-Max scaling preprocessing (0.93 and 0.92 of area under the curve, respectively). However, it is observed that the Median Filter method smooths sharp spectral features, resulting in high variability in the classification performance. Therefore, based on these results, obtained with different databases acquired by different HSI instrumentation, the most relevant preprocessing technique identified in this work is Min-Max scaling.

## 1. Introduction

In 2020, the World Health Organization (WHO) estimated that around 10 million deaths were caused by cancer, where the most common were lung, colon and rectum, liver, stomach, and breast cancers [1]. Physicians usually perform aggressive treatments including surgery, radiotherapy, and chemotherapy. Innovative methods are still needed to improve the diagnosis of tumor tissue during screening and treatment of the disease.

Hyperspectral (HS) Imaging (HSI) is a technology which combines spectroscopy and conventional imaging with the aim of obtaining simultaneously the spatial and the spectral information of a specific object [2]. HSI can capture a very large spectral range, from the ultraviolet to the infrared, providing abundant information that covers hundreds of spectral bands (also called spectral channels) for each pixel of the image [3]. HS camera sensors measure the light reflected, absorbed, and scattered by materials when they are illuminated by a light source. The reflectance measured at one pixel consists of an almost continuous spectrum that acts as a fingerprint (the so-called spectral signature), which can be employed to characterize the chemical composition of the captured material in such a pixel. The HS camera systems perform spatial or spectral scanning (depending on the type of HSI technology employed) and produce three-dimensional (3D) HS data with spatial and spectral components (also called an HS cube) [4,5]. The principal advantages of this technique are that it is non-ionizing, non-contact, non-invasive, and label-free. For these reasons, HSI is an emerging technique in the medical field [6]. It has been evaluated in many different applications, such as histopathological or sample analysis [7,8], wound care [9], retinal imaging [10], microcirculatory alterations [11], and tissue oxygen saturation monitoring [12]. Furthermore, in recent years, HSI combined with Artificial Intelligence (AI) analysis algorithms has shown promising results for cancer detection [13,14,15,16,17]. Several types of cancer have been investigated using in vivo and ex vivo tissue samples, such as skin cancer [18], head and neck cancer [19,20], breast cancer [21,22], and brain cancer [23,24,25,26].

However, unpredictable physical perturbations can cause additive, multiplicative, and wavelength-dependent scattering effects, altering the efficiency of the estimation by AI models [27]. To avoid or reduce this issue, it is recommended to preprocess the HS data. One of the most common preprocessing techniques in signal and image processing is the application of spectral range normalization, such as Min-Max scaling and Standard Normal Variate (SNV) normalizations. In the medical field, normalization preprocessing is also performed when considering HS data of different body areas and patients, when the data are acquired with different HSI systems under different external conditions. In general, this is achieved through calibration of the devices [28,29,30] and by applying SNV normalization [31,32]. There is so far no research which evaluates and recommends preprocessing methods and measures their influences on tissue classification tasks. Therefore, this work aims to evaluate different combinations of preprocessing techniques: (i) only calibration; (ii) the Gaussian filter; (iii) Min-Max scaling normalization; (iv) SNV normalization; and (v) a median filter in the spatial range. This evaluation is performed with the goal of achieving an improvement in tumor detection in three different use cases where HS data have been captured in previous work (i.e., colorectal [14], esophagogastric [33], and brain [34] cancers). One Machine Learning (ML) model, the Support Vector Machine (SVM), and one Deep Learning (DL) model, a 3D Convolutional Neural Network (3DCNN), were selected to perform the pixel-wise classification. Both models have shown promising results in previous studies ([23,33]). 

The main goal of this work is to identify the most relevant preprocessing method for independent medical HS databases, enabling a better classification and identification of tissues affected by the different types of tumors. 

## 2. Materials and Methods

In this section the three HS databases (colon, esophagogastric, and brain) employed will be described, as well as the proposed preprocessing and classification methods. In addition, the evaluation metrics used to evaluate the results are presented.

### 2.1. Data Acquisition

#### 2.1.1. Colon and Esophagogastric Cancer Databases

The colon and esophagogastric databases are composed of 12 and 10 patients, respectively, where all the patients were previously treated with chemo- and radiotherapy. For each patient, a HS cube was acquired using the commercial push-broom TIVITA^®^ Tissue device (Diaspective Vision GmbH, Am Salzhaff, Germany) which covers the spectral range from 500 to 1000 nm. The HSI images have a spectral resolution of 5 nm. The spatial resolution is 130 µm/pixel for a distance between tissue and camera of 50 cm. The acquisition time was approximately 10 s, and the spatial size of the image was 640 × 480 pixels. The light source includes six halogen spots of 20 W each. The study was approved by the local ethics committee of the medical Faculty of the University of Leipzig (026/18-ek) and was registered at Clinicaltrials.gov (NCT04230603). The HS data visualize ex vivo tissue samples, captured under standardized conditions during the surgical procedure within 5 min after resection. The lights in the operation room were switched off and the HS camera was positioned at the calibrated distance (~50 cm from the sample). 

**Figure 1:**
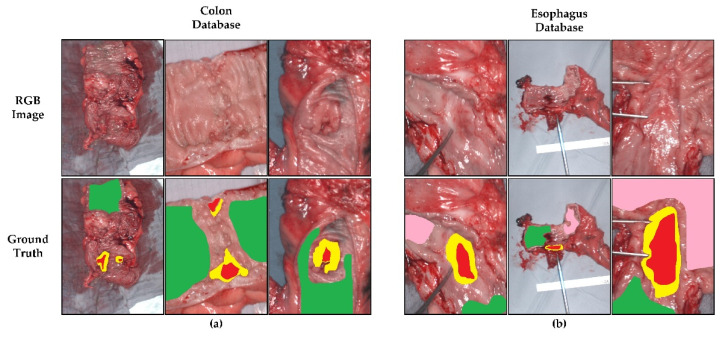
RGB images and ground truth from the databases acquired using the TIVITA^®^ Tissue system. (**a**) Colon database. (**b**) Esophagogastric database. Green color indicates healthy colon and esophagus tissue, pink color indicates healthy stomach tissue, red color indicates tumor tissue, and yellow color indicates tumor margin area.

For the region of interest (ROI) selection, the pathologist digitally segmented the tumors in the RGB image of the resected tissue samples using software (Gimp v.2.10). Additionally, an ROI including areas with high probability of healthy tissue was selected. The RGB images were reconstructed based on the HSI images. Therefore, the image sizes were the same and the spectral data corresponding to the ROIs were easy to extract. The pathologist performed the annotations of this database because she had experience with the visual recognition of tumors and healthy tissue. This skill is necessary for the pathologist to perform histological slices of the tumor [33]. With respect to the colon database, two classes were labelled: malignant tissue and colon tissue. Regarding the esophagogastric database, three classes were labelled: malignant tissue, stomach tissue, and esophagus tissue. Figure 1 depicts some examples from both databases. The first row shows the synthetic RGB images generated from the HS data and the second row shows the ground truth, where green indicates healthy colon and esophagus tissue, pink indicates healthy stomach tissue, red indicates tumor tissue, and yellow indicates tumor margins. However, the last class (margin) will not be evaluated in the proposed classification scheme since it can include both healthy and/or pathological tissues. 

#### 2.1.2. Brain Cancer Database

The system employed for the acquisition of the brain database was developed during a European research project called HELICoiD [24]. The system was composed of a HS push-broom camera manufactured by HeadWall Photonics, Hyperspec^®^ VNIR A-Series model. The spectral range covered from 400 to 1000 nm, capturing 826 spectral bands. This type of camera acquires the spectral information of a single narrow spatial line. The maximum spatial size of the HS image, considering the scanning platform, is 1004 × 1787 pixels, enclosing an area of 129 × 230 mm with a pixel size of 128.7 µm. The illumination system comprises a Quartz–Tungsten–Halogen (QTH) lamp connected to a cold light emitter allowing cold illumination over the brain surface. The HS data were obtained in vivo during neurosurgical procedures [34]. 

The ground truth annotation was performed using a methodology which combined the pathology assessment with neurosurgical criteria using the Spectral Angle Mapper (SAM) algorithm [34]. The labelled pixels are obtained by using a customized labelling tool developed for this purpose. First, the surgeon selects a reference pixel from the image for which she is sure that it corresponds to a certain type of tissue, e.g., normal tissue, tumor tissue, or hypervascularized areas. Once the reference pixel is selected, the SAM algorithm is applied to the remaining pixels in the HS image to determine which are spectrally similar to the reference pixel, and so are likely to belong to that class. Finally, the surgeon manually modifies a threshold to include or exclude pixels similar to the reference pixel, and to visually ensure that the selected pixels truly belong to the target class. This procedure is explained in more detail in [34]. Regarding the tumor tissue, the label was confirmed by the pathologist after the biopsy. Four different classes were defined: healthy brain tissue, tumor tissue, hypervascularized tissue (mainly blood vessels), and background. The background class involves the elements which are present in the images but are not relevant for the identification of tumor tissue within the brain (i.e., skull bone, dura, skin, or surgical materials).

**Figure 2:**
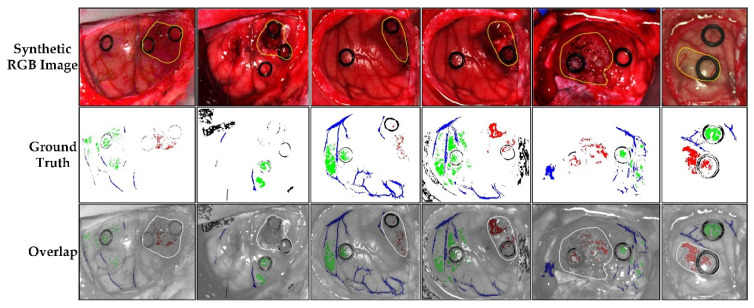
Synthetic RGB images and ground truths of six HS images captured from patients affected by grade IV glioblastomas within the brain cancer database. Green color indicates healthy tissue, red indicates tumor tissue, blue indicates hypervascularized tissue, and black indicates background. The yellow circles indicate the location of the tumor.

The brain database is composed of a total of 26 HS cubes belonging to 16 patients. Four patients (six HS images) suffered from a grade IV glioblastoma tumor. The remaining patients were affected by other types of tumors or underwent surgery due to other diseases. From these remaining patients, only healthy brain and hypervascularized tissue, as well as background, were used. The study protocol and consent procedures were approved by the Comité Ético de Investigación Clínica-Comité de Ética en la Investigación (CEIC/CEI) of the University Hospital of Gran Canaria Doctor Negrin (CEIC code: 130069). Figure 2 shows the six HS images captured from subjects affected by grade IV glioblastomas within this database, where the first row displays the synthetic RGB images, the second row shows the ground truth with the four classes, and the third row illustrates the overlap of the ground-truth with a grey scale image. Healthy tissue is represented in green color, tumor in red, hypervascularized tissue in blue, and background in black. 

#### 2.1.3. Summary Databases

Table 1 describes the three databases used, indicating the number of patients and the HS data, as well as the number of labeled pixels per class. The colon database includes two classes: tumor tissue (TT) and healthy colon tissue (CT). The esophagogastric database includes three classes: tumor tissue (TT), healthy esophagus tissue (ET), and healthy stomach tissue (ST). The brain database includes four classes: tumor tissue (TT), healthy brain tissue (BT), blood vessel (BV), and background (BG). It is worth noticing that the data are clearly unbalanced.

### 2.2. Processing Frameworks

**Figure 3:**
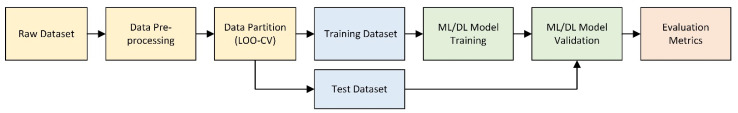
Generic processing-chain block diagram for classification using ML and DL models.

The different preprocessing methods were evaluated in the context of a tissue-classification task. The overall processing chain is depicted in Figure 3 and described in the following sections. 

#### 2.2.1. HS Data Calibration

The acquired raw HS data were calibrated to remove the lighting noise and the effects of dark currents from the HS sensors, according to Equation (1), where C is the calibrated HS data, R is the raw HS data, and $D_{ref}$ and $W_{ref}$ are the dark and white reference HS data, respectively. The white reference image was acquired using a material which reflects 99% of the incoming light. The dark image reference was acquired by keeping the camera shutter closed.

(1) $$C=100\cdot \frac{R-D_{ref}}{W_{ref}-D_{ref}}$$

#### 2.2.2. HS Data Preprocessing

**Figure 4:**
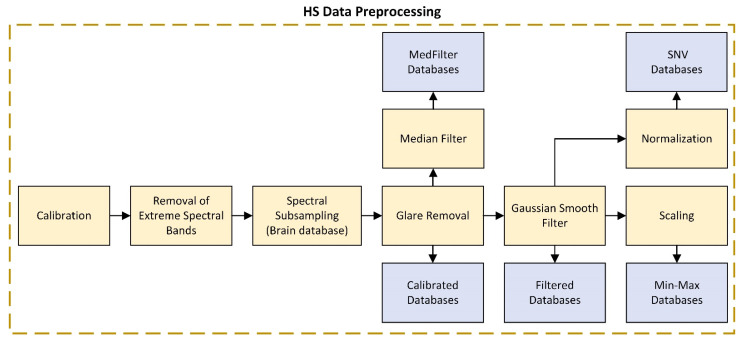
Steps of the preprocessing chain block diagram.

The data preprocessing applied to the calibrated data consisted of several steps shown in Figure 4 and described below. 

First, the spectral bands at the edges of the spectral range were removed because they included noise due to the low performance of the sensors for these wavelengths. Therefore, the filtered spectral ranges were 550–950 nm for the colon and esophagogastric databases, and 440–902 nm for the brain database. 

The second step was only applied to the brain database. The spectral bands of the HS data were sub-sampled from 826 to 128 with an interval step of 3.61 nm. It was demonstrated in [23] that by reducing the number of bands to 128, the overall classification accuracy was not significantly affected by the spectral subsampling; in fact, it was even proven that the sensitivity of the tumor classification was slightly higher than when employing the original data (826 bands) as a consequence of some noise reduction. Additionally, with spectral subsampling the execution time showed a speed-up factor of ~48× with respect to the classification time of the original data.

The third step consisted of removing the glare from the HS data of the colon and esophagogastric databases. First, we manually selected a threshold to identify the glare pixels. Then, the techniques of erosion and dilation were employed to allow the removal of pixels with small white noise, as well as those in their vicinity, to avoid outliers.

The fourth step involved filtering the noise in the spatial and spectral ranges independently. A Gaussian smoothing filter was applied in the spectral range for Min-Max scaling and SNV HS data preprocessing, while a median filter was applied in the spatial range for the MedFilter HS data preprocessing. 

The last step was the normalization of the HS data using the Min-Max scaling and SNV methods. Min-Max scaling is a technique in which a pre-defined boundary is set, usually in the range [0, 1], where 0 is the lowest value and 1 is the highest value of a pixel in the HS cube, as can be seen in Equation (2), where $C_{i}$ is the calibrated pixel of the HS cube. The SNV method consists of subtracting the mean spectral value from each spectral signature and dividing it by its own standard deviation; see Equation (3), where $C_{i,j}$ is the calibrated HS pixel, i represents the pixel’s elements, j represents the spectral bands, ${\bar{C}}_{i}$ is the mean value of the spectral signature of the pixel i, and L is the number of spectral bands [35]. Steps 1 to 3 were always performed according to the database used. We have tested the following combinations of preprocessing, including noise filtering and data scaling/normalization (steps 4 and 5): (1) no noise filtering or scaling/normalization (noted “Calibrated” in the following); (2) spatial and spectral smoothing (“Filtered”); (3) spectral smoothing and Min-Max scaling (“Min-Max”); (4) spectral smoothing and SNV normalization (“SNV”); and (5) spatial smoothing and normalization (“MedFilter”). The normalization performed in the median filter was SNV since, as discussed in Section 1, it is one of the most widely used standardizations for HS data in the medical field.

(2) $$C_{Min-Max}{}^{\prime }=\frac{C_{i}-min\left(C\right)}{\max\left(C\right)-min\left(C\right)}$$

(3) $$C_{i,j}^{SNV}{}^{\prime }=\frac{\left(C_{i,j}-{\bar{C}}_{i}\right)}{\sqrt{\frac{\sum_{j=1}^{L}{\left(C_{i,j}-{\bar{C}}_{i}\right)}^{2}}{L-1}}}$$

**Figure 5:**
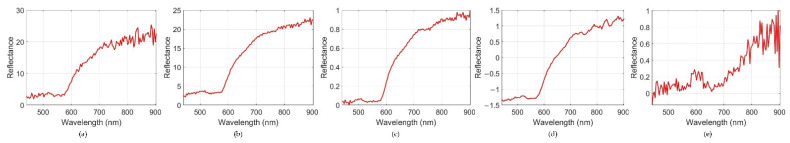
Spectral signature after applying different preprocessing techniques. (**a**) Calibrated preprocessing technique; (**b**) Filtered preprocessing technique; (**c**) Min-Max preprocessing technique; (**d**) SNV preprocessing technique; (**e**) MedFilter preprocessing technique.

Figure 5 shows that the signature of a pixel’s changes depending on the preprocessing technique employed. It can be observed that, except for MedFilter, the spectral signature of the same structure looks homogeneous.

#### 2.2.3. Summary Data Preprocessing

Table 2 summarizes each of the preprocessing methods to be evaluated in this work. Calibrated data are those without any preprocessing, where only the extreme spectral band removal step (to avoid the noise generated by the HS sensors in such bands, due to low performance) and the glare removal step (to avoid including these pixels in the classification step) have been applied. As for the Filtered data, a Gaussian filter is applied for noise reduction. Min-Max scaling and SNV data are normalized to obtain a more uniform spectrum. Regarding the MedFilter data, a median filter is applied to homogenize pixels having different intensities. 

#### 2.2.4. Machine Learning (ML) Model

SVM is the traditional ML model employed for the classification of HS data. It was successfully evaluated in previous work on the brain database [23]. SVM has been demonstrated to obtain remarkable results for highly unbalanced datasets [36]. It is a binary classification algorithm, which aims to find a hyperplane capable of separating the data with the widest possible margin [37]. The SVM implementation was carried out using MATLAB 2021a, using the linear kernel and a 0.5 classification threshold. Concerning the hyperparameters, the box constraints, cost, and weights were set to 1, and the prior probability was activated. This model was computed on an Intel(R) Core (TM) i7-4790K CPU (Central Processing Unit). 

#### 2.2.5. Deep Learning (DL) Model

The 3DCNN is a DL algorithm which has obtained significant results using unbalanced data [38]. CNNs work by dividing the HS cube into sub-cubes, called patches, where each of them is processed independently. The 3DCNN employed was developed by Hamida et al. [39]. They designed four different models; their model D is employed in this study, with an architecture of 8 layers and 5 × 5 networks. The inputs of the model are patches of the HS data with a size of 5 × 5 × *L*, where *L* is the number of spectral bands. Inspired by SqueezeNet [40], the 3DCNN was decomposed into three sequences of 1DCNNs (1D Convolutional Neural Networks), where the last layer was connected with *N* output neurons, *N* being the number of classes. In the case of the colon database, optimal thresholds were calculated for two classes, while for the multi-class databases (esophagogastric and brain databases) a default threshold was employed, as can be observed in Table 3. These values are obtained due to the imbalance of the dataset. However, they also preserve a balance between sensitivity and specificity. This model was already successfully evaluated on the colon and esophagogastric databases [33], and it was implemented in Python 3.7 using the Tensorflow 2.4 and Keras 2.3 libraries on an AMD EPYC 32 core processor CPU and 2 RXT2080TI GPUs.

#### 2.2.6. Data Partition

A leave-one-out cross-validation was performed. This method performs an inter-patient classification, where the samples of an independent patient are used for the test, while the training group includes all the patients’ samples except the ones to be tested. In addition, for the ML approach, a reduction of the training dataset was performed to shorten the high execution time per patient. Martinez et al. [23] demonstrated that in some cases, the labelled data may contain redundant information, increasing the execution time and even worsening the classification performance. This study showed that for the brain cancer database, by reducing the training dataset to 1000 pixels per class, the performance results were equivalent to or slightly better than using all the labelled pixels. Moreover, the execution time involved for the training of the SVM model was drastically reduced. The data-reduction methodology was based on the employment of the K-Means algorithm, which was applied in each group of labelled pixels (each class) with the objective of obtaining 100 different clusters per labelled group. Thus, 100 centroids that relate to a specific class were obtained. To reduce the training dataset, such centroids were used to recognize the most representative pixels of each class by using the SAM algorithm [41]. For each centroid, the ten most similar pixels were selected, obtaining a total of 1000 pixels per class. 

In this study, the number of clusters employed remains the same as in [23], i.e., 100 clusters per class. However, the number of similar pixels per class has been adapted according to the evaluated database. With respect to the colon database, 10 pixels per centroid were initially selected, having a total of 1000 pixels per class. The results were not sufficiently efficient, so 20 pixels per centroid were selected, obtaining 2000 pixels per class, 4000 in total. The same applied to the esophagogastric database: the results were not good enough when selecting only 10 pixels per centroid, so 20 pixels per centroid were selected instead, obtaining a total of 6000 pixels for the three classes. Finally, for the brain database we employed the same numbers as in [23], 10 pixels per centroid, obtaining 1000 pixels per class and a total of 4000 pixels. In this case it was not necessary to increase the number of pixels per centroid since in [23] it is demonstrated that with this number of pixels, good results are obtained.

#### 2.2.7. Evaluation Metrics

Since the test dataset was unbalanced in each database, the following quantitative evaluation metrics were selected: F_1_-Score, Area Under the Curve (AUC), and Matthew Correlation Coefficient (MCC). For a qualitative evaluation of the results, classification maps and probability maps were generated and visualized. Finally, paired two-tailed t-tests were employed to determine whether the difference in the mean AUC of the models (SVM and 3DCNN) was significantly different.

F_1_-Score is the harmonic mean of precision and recall. It is defined by Equation (4), where *TP* represents the true positives, *FP* the false positives, and *FN* the false negatives. The AUC was computed based on the Receiver Operating Characteristic (ROC) curve. The MCC is defined by Equation (5), where *TN* represents the true negatives. This metric computes the correlation coefficient between the observed and the predicted values, used to analyze unbalanced data. MCC has a value range between [−1, 1]. The extreme values represent a completely wrong and completely correct prediction, respectively.

(4) $$F_{1}\text{-}\mathrm{Score}=\frac{2TP}{2TP+FP+FN}$$

(5) $$\mathrm{MCC}=\frac{TP\cdot TN-FP\cdot FN}{\sqrt{\left(TP+FP\right)\cdot \left(TP+FN\right)\cdot \left(TN+FP\right)\cdot \left(TN+FN\right)}}$$

The classification maps visualize the predicted labels. A color is assigned to each pixel belonging to a specific predicted class. For the colon database, green represents the colon tissue and red the tumor tissue. In the esophagogastric database, green was selected to represent the esophagus class, red the tumor class, and pink the stomach class. Lastly, for the brain database, green was assigned to the normal tissue, red to the tumor tissue, blue to the blood vessels, and black to the background. As for the probability maps, the colors indicate the probability that the pixel belongs to the class under evaluation. In this case, the maps show the probability of the pixels belonging to the tumor class. Red color indicates the higher probability (1) of belonging to the tumor class, while blue color refers to the lower probability (0) of belonging to the tumor class.

With respect to the paired two-tailed t-test, this statistical method is employed to evaluate whether the difference in the mean AUC of the models was significantly different (SMV vs. 3DCNN). The *p*-value is considered statistically significant if *p* is lower than 0.05 (p≤ 0.05).

## 3. Results

### 3.1. Colon Results

The performance results of the models are shown in Table 4. As can be seen, the most accurate results were obtained with the Calibrated 3DCNN, the Filtered 3DCNN, and the Median Filter 3DCNN models. For the tumor class, the Calibrated 3DCNN and the MedFilter 3DCNN both achieved an F_1_-Score of 0.52, the Filtered 3DCNN reached an AUC of 0.96, and the MedFilter 3DCNN obtained an MCC of 0.52. For the colon class, the best performance was obtained with the MedFilter SVM with an F_1_-Score of 0.98. The results showed that the MedFilter preprocessing clearly improved the performance of both the SVM and 3DCNN models. With respect to the paired two-tailed *t*-test (SVM vs. 3DCNN), a significant difference can be observed in the mean AUC of the models (p=0.003 for Calibrated and Filtered, p=0.007 for Min-Max and SNV, and p=0.04 for MedFilter). 

**Figure 6:**
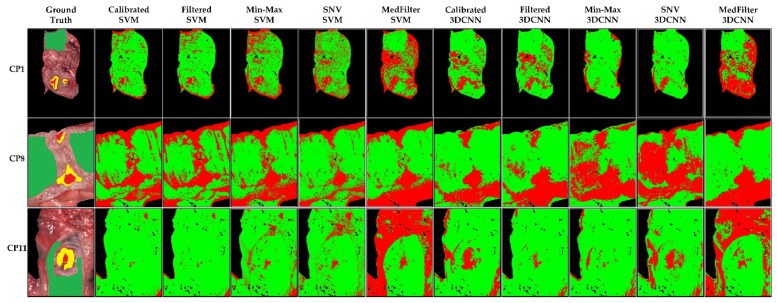
Classification maps of three different patients from the colon database, as computed by each model. Green represents healthy colon tissue, red tumor tissue, and yellow tumor margin area.

The classification maps in Figure 6 show that the tumor class was correctly identified, except for the Min-Max and SNV 3DCNN model. In general, the labeled areas are correctly identified. However, the classification of the rest of the image varies between models. This can be visualized in patient CP11, where the unlabeled tissue was mainly classified as healthy tissue. However, when the MedFilter was used, it was identified as tumor. On these three examples, the Calibrated 3DCNN provided a better visual delineation of the labeled structures. Figure S1 in the Supplementary Material shows the classification maps for the remaining patients included in the test dataset.

**Figure 7:**
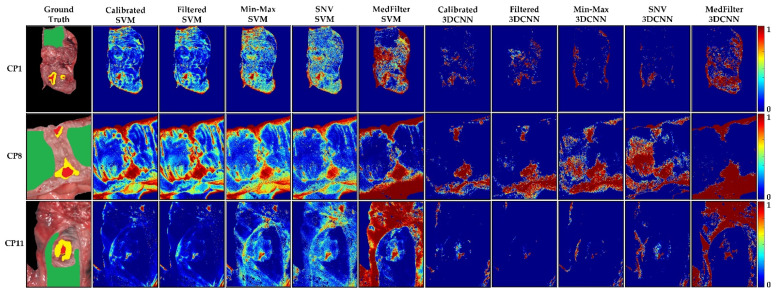
Tumor class probability maps in three different patients (CP1, CP8, and CP11) from the colon database. The red color indicates a high probability to correspond to the tumor class (1), while the blue color indicates a low probability to correspond to the tumor class (0).

Probability maps for whether individual pixels belong to the tumor class are depicted in Figure 7. In general, the maps show a high probability in the area corresponding to the annotated tumor. However, for non-labeled areas, a large number of false positives is observed. Explanations could be that tissues are different from the labeled classes (deeper tissues) or they could be cauterized during the surgery, among other factors. Considering only the labeled tumor pixels, the MedFilter SVM and 3DCNN models achieved an accurate delineation of the tumor area, which is consistent with the quantitative results. Figure S2 in the Supplementary Material shows the probability maps of each patient in the test dataset. 

### 3.2. Esophagogastric Results

Table 5 shows the classification results obtained for the esophagogastric database. The models with the best performance with respect to the F_1_-Score are the Min-Max SVM and MedFilter SVM, with 0.58 for the tumor class. With respect to the AUC, the Min-Max SVM model achieved 0.93 for the tumor class, and 0.94 and 0.95 for the esophagus and stomach tissue classes, respectively. However, with respect to the MCC metric, the best results for tumor classification were obtained by the Calibrated 3DCNN model (0.51). The Min-Max model tended to improve the performance of the classification, while no preprocessing was required for the 3DCNN to obtain high performance. In this case, the paired two-tailed t-test was employed to evaluate whether the mean AUCs of the models have significant differences. It can be observed that Filtered models (SVM vs. 3DCNN) have a significant statistical difference (p=0.005). The remaining models obtained the following *p*-values: p=0.18 for Calibrated, p=0.17 for Min-Max, p=0.14 for SNV, and p=0.06 for MedFilter. 

**Figure 8:**
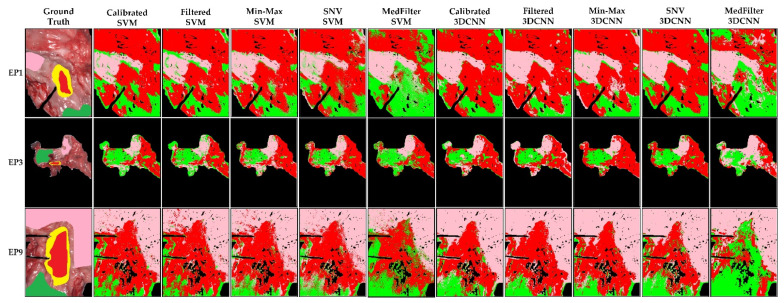
Classification maps of three different patients (EP1, EP3, and EP9) from the esophagogastric database, as computed by each model. Green represents healthy esophagus tissue, red tumor tissue, pink healthy stomach tissue, and yellow tumor margin area.

Figure 8 shows the classification maps, where we can observe that, except for the MedFilter models, the maps look very similar. In EP1, no model can clearly identify the center of the tumor, with the Calibrated 3DCNN model being the most accurate. In the case of EP3, the MedFilter SVM model provided a better delineation of the different classes. It showed a reduced rate of misclassification of the tumor class, relative to the esophagus class. Finally, for EP9, the Min-Max 3DCNN model provided the best visual classification. Figure S3 in the Supplementary Material illustrates the classification maps of the remaining patients of the test dataset.

**Figure 9:**
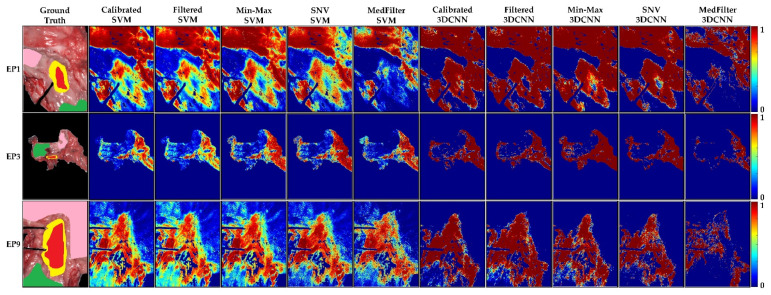
Tumor class probability maps in three different patients (EP1, EP3, and EP9) from the esophagogastric database. The red color indicates a high probability to correspond to the tumor class (1), while the blue color indicates a low probability to correspond to the tumor class (0).

The probability maps in Figure 9 show that for EP1, only the Min-Max and SNV 3DCNN models could correctly detect the tumor tissue. For EP3 and EP9, all models except the MedFilter 3DCNN model, correctly identified the tumor area. However, a large number of false positives is observed in the image. With respect to the SVM model, the Min-Max and SNV preprocessing provided a better delineation of the tumor. In the Supplementary Material, Figure S4 shows the probability maps of the remaining patients included in the test dataset.

### 3.3. Brain Results

In Table 6, the classification performance of the different models and preprocessing methods are shown. With respect to the F_1_-Score, it can be observed that the SVM models obtained better results (0.67 to 0.82) than the 3DCNN models (0.41 to 0.52) for tumor classification. In the case of the AUC metric, the best result with respect to the tumor class was obtained by the Filtered SVM model with a value of 0.94, followed by the Calibrated SVM and Min-Max SVM models with values of 0.92. Lastly, considering the MCC metric, the best-balanced model is the Filtered SVM, achieving 0.61 of the tumor class. Although the values of the metrics between the different preprocessing methods and classification models are relatively close, spatial and spectral smoothing tended to improve the classification performance of the SVM model, while Min-Max scaling tended to improve that of the 3DCNN. With respect to the *p*-values obtained with the paired two-tailed t-test, no significant difference was observed in the mean AUC between the models (p=0.48 for Calibrated, p=0.18 for Filtered, p=0.50 for Min-Max, p=0.18 for SNV, and p=0.12 for MedFilter).

**Figure 10:**
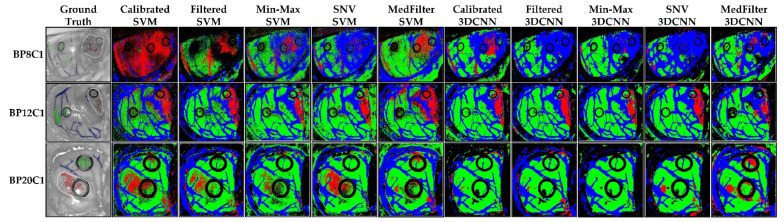
Classification maps of three different patients (BP8C1, BP12C1, and BP20C1) from the brain database, as computed by each model. Green represents healthy brain tissue, red tumor tissue, blue blood vessels, and black the background.

Figure 10 shows the classification map of three different patients. In the case of BP8C1, only the MedFilter SVM model was able to detect the tumor. The other classes were well-classified by all models, except the Calibrated SVM. For BP12C1, all models correctly identified the tumor class, and the Min-Max 3DCNN and SNV 3DCNN models clearly provided structure delineations. For BP20C1, the tumor area was correctly identified by the SVM model only. In general, the Filtered SVM model best visualized the tumor class of patients. Figure S5 in the Supplementary Material shows the classification maps of each patient in the test dataset.

**Figure 11:**
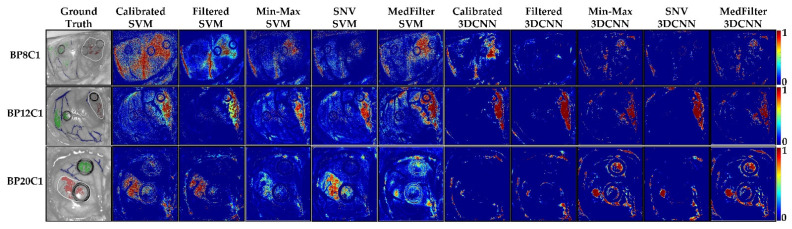
Tumor class probability maps in three different patients (BP8C1, BP12C1, and BP20C1) from the brain database. The red color indicates a high probability to correspond to the tumor class (1), while the blue color indicates a low probability to correspond to the tumor class (0).

Regarding the probability maps shown in Figure 11, it can be observed that in the case of BP8C1, only the Min-Max SVM, MedFilter SVM, and Calibrated 3DCNN models identified parts of the tumor area. For BP12C1, all models identified the tumor area with a high probability. Only the MedFilter SVM model showed more false positives. For BP20C1, the Calibrated, Filtered, and SNV SVM models were able to identify the tumor area. With respect to the SVM model, the Filtered, Min-Max, and SNV preprocessing provided a better delineation of the tumor. Figure S6 in the Supplementary Material visualizes the probability maps of the remaining patients included in the test dataset.

## 4. Discussion

This study reveals the importance of data preprocessing steps for tissue segmentation purposes in the medical HSI field. Depending on the type of HS camera and the different tissue type to be evaluated, it is necessary to carry out a preliminary study analyzing which preprocessing technique is the most suitable in each case. In the case of the colon and esophagus databases, the HS data were captured by the TIVITA^®^ Tissue system, while the brain database was captured by the HELICoiD demonstrator. 

For the colon database, all the models were statistically significant (p=0.003 for Calibrated and Filtered, p=0.007 for Min-Max and SNV, and p=0.04 for MedFilter), indicating in this case that the type of model employed has no relevance. However, the optimal combination of preprocessing and classification model was, for both quantitative and qualitative results, the MedFilter 3DCNN model, achieving an F_1_-Score of 0.52 for the tumor class. Additionally, the MedFilter clearly also improved the performance of the SVM. For the esophagogastric database, only Filtered models have a significant statistical difference, p=0.005. The SVM models provided the best quantitative and qualitative results with respect to tumor tissue. F_1_-Scores of 0.58 for the identification of the tumor class were obtained with Min-Max and MedFilter preprocessing. The performance of the 3DCNN models for tumor detection was higher with the calibrated preprocessing, with an F_1_-Score of 0.53. The rest of the models showed a lower performance, with a tumor class F_1_-Score of 0.45. However, the best results for the MCC metric were obtained with the Calibrated 3DCNN model. This was confirmed by the qualitative results. For the brain database, no significant statistical difference was obtained, and it was more difficult to select the best preprocessing method. The SVM model provided the best results for tumor classification with F_1_-Scores of 0.82 and 0.80 for Filtered and Min-Max preprocessing, respectively. This fact was confirmed by the visualization of the classification and probability maps. The 3DCNN model achieved an F_1_-Score of 0.52 with respect to the tumor class. Filtered and Min-Max preprocessing tended to improve the performances of the SVM and 3DCNN classification models, respectively.

It was observed in this study that only by performing the filtered step to standardize the illumination conditions could similar values to the best results obtained be achieved. Furthermore, with respect to the two-tailed paired *t*-test, it was observed that the best *p*-values were obtained by Filtered preprocessing in each of the databases (p=0.003, p=0.005, and p=0.18). However, due to the classification models, saturation can occur when there are values greater than 1 or −1. Regarding the different kinds of preprocessing, it is necessary to highlight how each method can affect the spectral features and the subsequent classification. First, it can be observed that Min-Max is greatly affected by the extreme values (minimum and maximum values). SNV reduces the variations between the absolute reflectance values, enabling us to compare HS data of different patients acquired under different external conditions. However, SNV can smooth sharp spectral features, which can reduce the classification performance if those spectral features were relevant. The same also applies when the median filter is performed, as it tends to smooth out the values that have a large difference from their neighbors, distorting the data. 

Different conclusions can be deduced based on this study: first, it is necessary to carry out a preliminary study analyzing which preprocessing technique is the most suitable, depending on the type of HS camera and the different tissues to be evaluated. This can be observed with patient BP20C1 of the brain database. Previous segmentation performed without preprocessing steps led to the non-identification of the tumor area using ML techniques [23,25]. Second, statistical results show that the results are worse when more than two classes are taken into account. Third, the probability and classification maps showed that the annotated tumor tissue was in general correctly identified by the two-classification model tested. On the other hand, these maps also show a remarkable number of false positives. This may be due to several reasons. One reason could be the spatial resolution of the data. Since the classification is performed pixel-wise, having a small pixel size (high resolution sensor) assumes that one pixel includes only one type of tissue. In low spatial resolution data, one pixel may include information from several tissues. The spectral signature of the pixel is the combination of the spectral signatures of the different tissue types, which may result in worsen classification results. In such a situation, spectral unmixing methods are more suitable to extract the different tissues represented in a pixel [42]. However, how the spatial resolution is affecting the quality of the classification should be further investigated in the future. Another reason is that the colon and esophagogastric databases are composed of ex vivo tissues. This can explain the lower performance in comparison to the results obtained with the brain database, which includes in vivo tissues. The tissue has been exposed to chemo- and radiotherapy, altering the tissue composition in the colon and esophagogastric databases. This leads to large inter-patient variabilities. A further reason is the limited number of available patients in the study. Training the models on larger datasets should increase the classification performance. 

## 5. Conclusions

HSI has proved to be a promising technology for identifying cancerous tissue. In this paper, the importance of carrying out an evaluation of different preprocessing techniques is highlighted, looking for the most appropriate one depending on the target application. As has been shown, this can vary depending on the type of tissue to be studied and the HS instrumentation employed. In this study, Min-Max scaling was shown to be one of the preprocessing steps which provides the best performance results. 

Future work will be carried out on an increasing the number of patients and classes, in order to evaluate the ML and DL models with more robustness. The possibility of using animal tissue has also been raised, increasing the number of subjects, and thus allowing the validation of the proposed models. 

## Figures and Tables

**Table 1:** Summary of the HS-labelled databases employed in this study.

Database	Patients	HS Cubes	#Labelled Pixels			
			TT	CT/ET/BT	ST/BV	BG
Colon	12	12	75,588	668,927	-	-
Esophagogastric	10	10	61,414	369,256	286,554	-
Brain	16	26	11,054	101,706	38,784	118,132

TT: Tumor Tissue; CT: Colon Tissue; ET: Esophagus Tissue; BT: Brain Tissue; ST: Stomach Tissue; BV: Blood Vessel; BG: Background.

**Table 2:** Summary of data preprocessing steps.

Name	Preprocessing Steps	Brief Comment
Calibrated	Extreme band removal + Glare removal	Calibration required for standardization of spectral signatures concerning equipment and illumination
Filtered	Calibrated + Gaussian smoothing filter	Application of a Gaussian filter for noise reduction in HS data
Min-Max	Filtered + Min-Max scaling	Reduces the range of values to [0, 1] to improve classification
SNV	Filtered + SNV normalization	SNV seeks to ensure that all spectra are comparable in terms of intensity. All spectra must have a mean of 0 and a standard deviation of 1.
MedFilter	Calibrated + Median filter spatial smoothing + SNV normalization	Homogenizes pixels with different intensities.

**Table 3:** Optimal threshold calculated for each database performed by 3DCNN.

Models		Colon Threshold	Esophagogastric Threshold	Brain Threshold
3DCNN	Calibrated	0.001	0.5	0.5
	Filtered	0.0037	0.5	0.5
	Min-Max	0.0189	0.5	0.5
	SNV	0.0028	0.5	0.5
	MedFilter	0.0081	0.5	0.5

**Table 4:** Mean and standard deviation values of the quantitative metrics obtained on the colon database according to different preprocessing and classification algorithms.

Models		F_1_-Score		AUC	MCC
		TT	CT		
SVM	Calibrated	0.36 ± 0.26	0.94 ± 0.12	0.69 ± 0.26 **	0.21 ± 0.27
	Filtered	0.38 ± 0.28	0.87 ± 0.25	0.68 ± 0.27 **	0.22 ± 0.27
	Min-Max	0.31 ± 0.26	0.97 ± 0.03	0.71 ± 0.23 *	0.19 ± 0.21
	SNV	0.32 ± 0.21	0.97 ± 0.03	0.74 ± 0.18 *	0.19 ± 0.21
	**MedFilter**	**0.43 ± 0.28**	**0.98 ± 0.03**	**0.84 ± 0.15** *	**0.36 ± 0.27**
3DCNN	Calibrated	**0.52 ± 0.21**	0.90 ± 0.11	0.94 ± 0.06 **	0.50 ± 0.19
	Filtered	0.46 ± 0.26	0.88 ± 0.11	**0.96 ± 0.03** **	0.48 ± 0.22
	Min-Max	0.38 ± 0.26	0.85 ± 0.12	0.93 ± 0.08 *	0.37 ± 0.22
	SNV 3	0.45 ± 0.22	0.87 ± 0.13	0.90 ± 0.08 *	0.44 ± 0.20
	**MedFilter**	**0.52 ± 0.23**	**0.91 ± 0.08**	0.94 ± 0.08 *	**0.52 ± 0.20**

TT: Tumor Tissue; CT: Colon Tissue, *: *p* < 0.05; **: *p* < 0.01.

**Table 5:** Mean and standard deviation values of the quantitative metrics obtained on the esophagogastric database according to different preprocessing and classification algorithms.

Models		F_1_-Score			AUC			MCC		
		TT	ET	ST	TT	ET	ST	TT	ET	ST
SVM	Calibrated	0.56 ± 0.32	0.91 ± 0.09	**0.90 ± 0.13**	0.91 ± 0.06	0.92 ± 0.05	**0.95 ± 0.11**	0.42 ± 0.21	0.60 ± 0.13	0.71 ± 0.30
	Filtered	0.53 ± 0.31	0.89 ± 0.10	0.89 ± 0.13	0.90 ± 0.06 **	0.89 ± 0.05	0.93 ± 0.11	0.40 ± 0.20	0.53 ± 0.11	0.62 ± 0.26
	**Min-Max**	**0.58 ± 0.30**	**0.96 ± 0.05**	0.84 ± 0.12	**0.93 ± 0.05**	**0.94 ± 0.05**	**0.95 ± 0.10**	0.41 ± 0.23	0.62 ± 0.12	0.64 ± 0.27
	SNV	0.56 ± 0.32	0.95 ± 0.06	0.86 ± 0.12	0.92 ± 0.05	0.93 ± 0.05	0.94 ± 0.11	**0.44 ± 0.23**	**0.64 ± 0.15**	0.68 ± 0.28
	MedFilter	**0.58 ± 0.35**	0.90 ± 0.11	0.88 ± 0.18	0.90 ± 0.08	0.93 ± 0.05	0.93 ± 0.13	0.41 ± 0.22	0.62 ± 0.19	**0.72 ± 0.32**
3DCNN	**Calibrated**	**0.53 ± 0.30**	**0.89 ± 0.05**	**0.82 ± 0.34**	0.90 ± 0.08	0.86 ± 0.22	**0.95 ± 0.11**	**0.51 ± 0.29**	**0.77 ± 0.11**	**0.79 ± 0.35**
	Filtered	0.42 ± 0.29	0.84 ± 0.07	0.79 ± 0.33	0.92 ± 0.06 **	0.90 ± 0.09	**0.95 ± 0.09**	0.42 ± 0.26	0.70 ± 0.12	0.75 ± 0.31
	Min-Max	0.44 ± 0.31	0.78 ± 0.12	0.77 ± 0.33	**0.92 ± 0.06**	0.82 ± 0.16	**0.95 ± 0.10**	0.44 ± 0.27	0.63 ± 0.15	0.73 ± 0.32
	SNV	0.45 ± 0.30	0.84 ± 0.07	0.80 ± 0.33	0.89 ± 0.09	0.83 ± 0.24	0.94 ± 0.11	0.42 ± 0.28	0.69 ± 0.13	0.76 ± 0.32
	MedFilter	0.33 ± 0.15	0.77 ± 0.13	0.79 ± 0.26	0.86 ± 0.12	**0.92 ± 0.08**	0.92 ± 0.15	0.34 ± 0.16	0.60 ± 0.22	0.72 ± 0.32

TT: Tumor Tissue; ET: Esophagus Tissue; ST: Stomach Tissue; **: *p* < 0.01.

**Table 6:** Mean and standard deviation values of the quantitative metrics obtained from the brain database according to different preprocessing and classification algorithms.

Models		F_1_-Score				AUC				MCC			
		BT	TT	BV	BG	BT	TT	BV	BG	BT	TT	BV	BG
SVM	Calibrated	**0.92 ± 0.09**	0.68 ± 0.27	**0.98 ± 0.02**	0.79 ± 0.20	0.90 ± 0.11	0.92 ± 0.07	0.96 ± 0.07	0.98 ± 0.02	0.58 ± 0.28	0.55 ± 0.26	0.81 ± 0.20	0.72 ± 0.20
	**Filtered**	**0.92 ± 0.16**	**0.82 ± 0.29**	0.97 ± 0.07	0.86 ± 0.23	0.95 ± 0.08	**0.94 ± 0.05**	0.97 ± 0.05	**1.00 ± 0.00**	0.75 ± 0.24	**0.61 ± 0.29**	**0.84 ± 0.17**	0.82 ± 0.23
	Min-Max	0.84 ± 0.14	0.80 ± 0.25	0.92 ± 0.12	0.88 ± 0.13	0.95 ± 0.05	0.92 ± 0.10	0.97 ± 0.05	0.98 ± 0.04	0.71 ± 0.17	0.55 ± 0.26	0.81 ± 0.21	0.79 ± 0.18
	SNV	0.87 ± 0.10	0.67 ± 0.32	0.90 ± 0.16	**0.97 ± 0.03**	**0.97 ± 0.03**	0.86 ± 0.20	0.96 ± 0.07	0.99 ± 0.02	**0.79 ± 0.11**	0.48 ± 0.39	0.78 ± 0.20	**0.88 ± 0.14**
	MedFilter	0.85 ± 0.17	0.78 ± 0.23	0.96 ± 0.06	0.83 ± 0.17	0.93 ± 0.07	0.90 ± 0.07	**0.98 ± 0.03**	0.98 ± 0.03	0.67 ± 0.22	0.52 ± 0.23	0.81 ± 0.07	0.80 ± 0.12
3DCNN	Calibrated	0.84 ± 0.19	**0.52 ± 0.40**	0.88 ± 0.12	0.97 ± 0.04	0.93 ± 0.13	0.86 ± 0.17	0.98 ± 0.04	**0.99 ± 0.02**	0.81 ± 0.21	0.51 ± 0.38	0.84 ± 0.15	**0.96 ± 0.05**
	Filtered	0.82 ± 0.19	0.37 ± 0.40	**0.89 ± 0.12**	0.96 ± 0.04	0.93 ± 0.13	0.79 ± 0.17	**0.99 ± 0.04**	0.98 ± 0.02	0.79 ± 0.21	0.37 ± 0.32	**0.86 ± 0.15**	0.94 ± 0.05
	**Min-Max**	**0.86 ± 0.20**	0.44 ± 0.44	0.87 ± 0.1	**0.98 ± 0.01**	**0.97 ± 0.04**	0.87 ± 0.14	0.97 ± 0.03	0.98 ± 0.03	**0.83 ± 0.23**	**0.54 ± 0.40**	0.83 ± 0.23	**0.96 ± 0.02**
	SNV	0.84 ± 0.19	0.44 ± 0.42	0.87 ± 0.14	0.97 ± 0.02	**0.97 ± 0.05**	**0.90 ± 0.10**	0.95 ± 0.09	0.96 ± 0.01	0.80 ± 0.22	0.45 ± 0.41	0.82 ± 0.18	**0.96 ± 0.03**
	MedFilter	0.75 ± 0.30	0.41 ± 0.33	0.86 ± 0.10	0.92 ± 0.09	0.78 ± 0.17	0.83 ± 0.34	0.98 ± 0.03	**0.99 ± 0.01**	0.68 ± 0.39	0.40 ± 0.36	0.82 ± 0.12	0.91 ± 0.09

TT: Tumor Tissue; BT: Brain Tissue; BV: Blood Vessel; BG: Background.

## Data Availability

All the brain HS image files generated from this study are available from: http://hsibraindatabase.iuma.ulpgc.es (accessed on 14 March 2019). Regarding the colon and esophagogastric databases, data can be obtained upon request at the following e-mail: claire.chalopin@medizin.uni-leipzig.de.

## Supplementary Materials

The following supporting information can be downloaded at: https://www.mdpi.com/article/10.3390/s22228917/s1, Figure S1. Classification map results of each model, for each patient in the colon database. Green color indicates colon, red color represents tumor tissue, and yellow represent the marginal area. Figure S2. Tumor class probability map results for each patient in the colon database. Figure S3. Classification map results of each model, for each patient in the esophagogastric database. Green color indicates healthy esophagus tissue, red color represents tumor tissue, pink color identifies healthy stomach tissue, and yellow represents the marginal area. Figure S4. Tumor class probability map results for each patient in the esophagogastric database. Figure S5. Classification map results of each model, for each patient in the brain database. Green color indicates healthy brain tissue, red color represents tumor tissue, blue color identifies the blood vessels, and black represents the background. Figure S6. Tumor class probability map results for each patient in the brain database.
