# Supplementary material for: Evaluation of Preprocessing Methods on Independent Medical Hyperspectral Databases to Improve Analysis
Source: Sensors (Basel). 2022 Nov 18;22(22):8917. doi: 10.3390/s22228917 (PMC9693077; doi:10.3390/s22228917)
Supplement: Supplementary file 1 [file sensors-22-08917-s001.zip › sensors-1993817-SI.pdf]

Evaluation of Preprocessing Methods on Independent Medical Hyperspectral Databases to Improve Analysis

Supplementary material

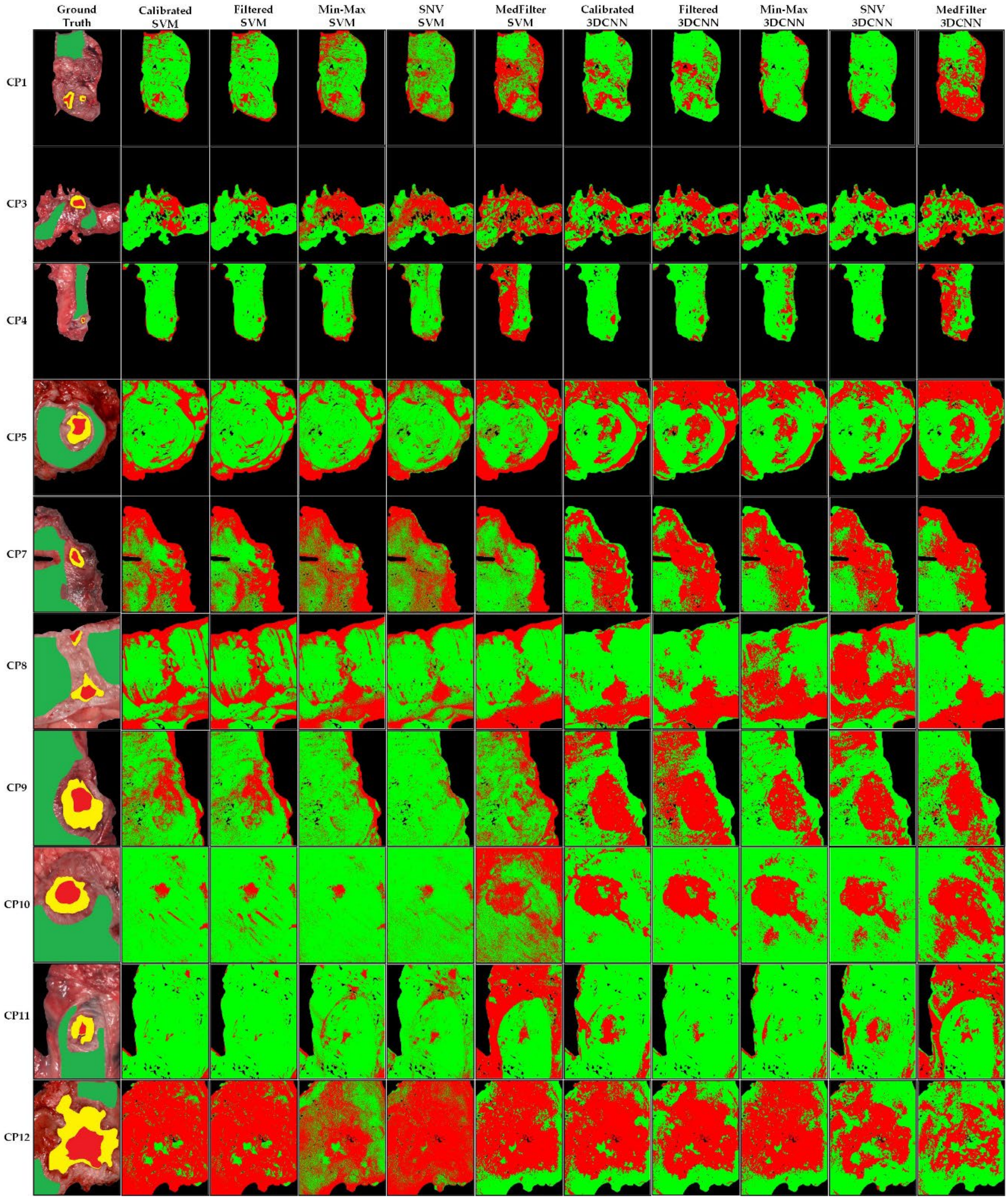

Figure S1. Classification maps results of each model of the colon database in each patient. Green color indicates colon, red color represents tumor tissue, and yellow represent the margin area.

Evaluation of Preprocessing Methods on Independent Medical Hyperspectral Databases to Improve Analysis

(Supplementary Material)

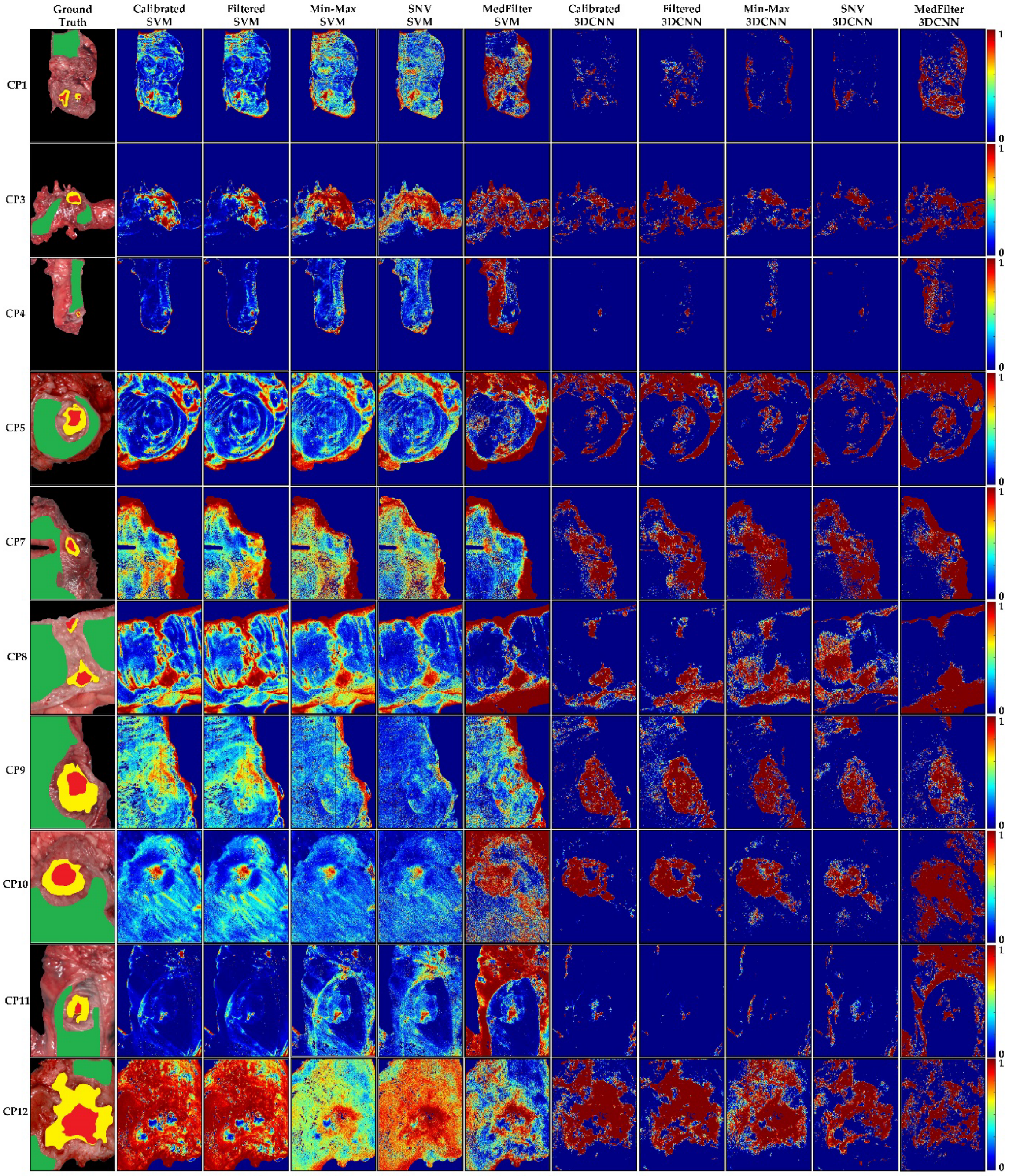

Figure S2. Tumor class probability maps results of the colon database in each patient.

Evaluation of Preprocessing Methods on Independent Medical Hyperspectral Databases to Improve Analysis

(Supplementary Material)

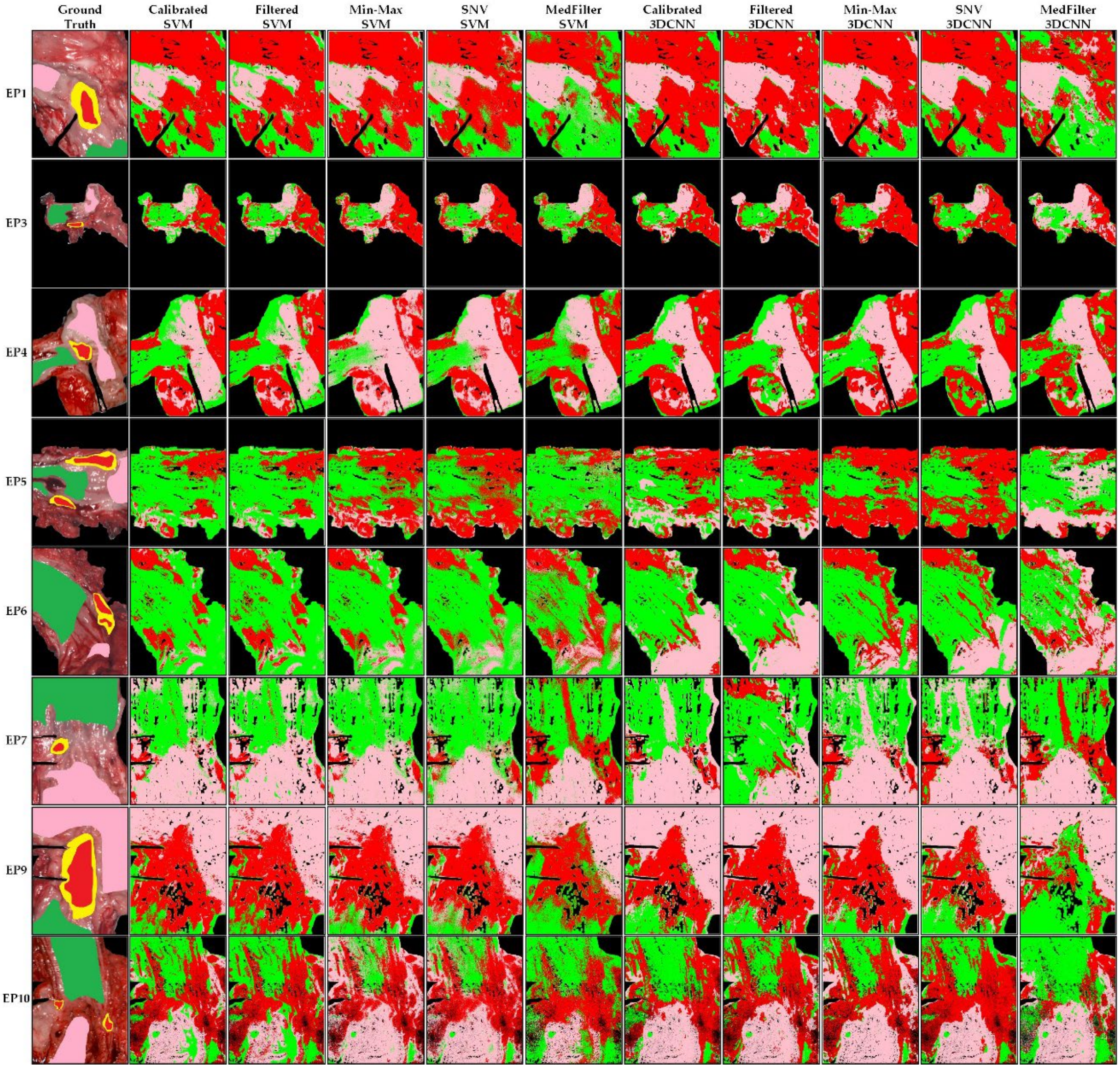

Figure S3. Classification maps results of each model of the esophagogastric database in each patient. Green color indicates esophagus healthy tissue, red color represents tumor tissue, pink color identifies the stomach healthy tissue and yellow represent the margin area.

Evaluation of Preprocessing Methods on Independent Medical Hyperspectral Databases to Improve Analysis

(Supplementary Material)

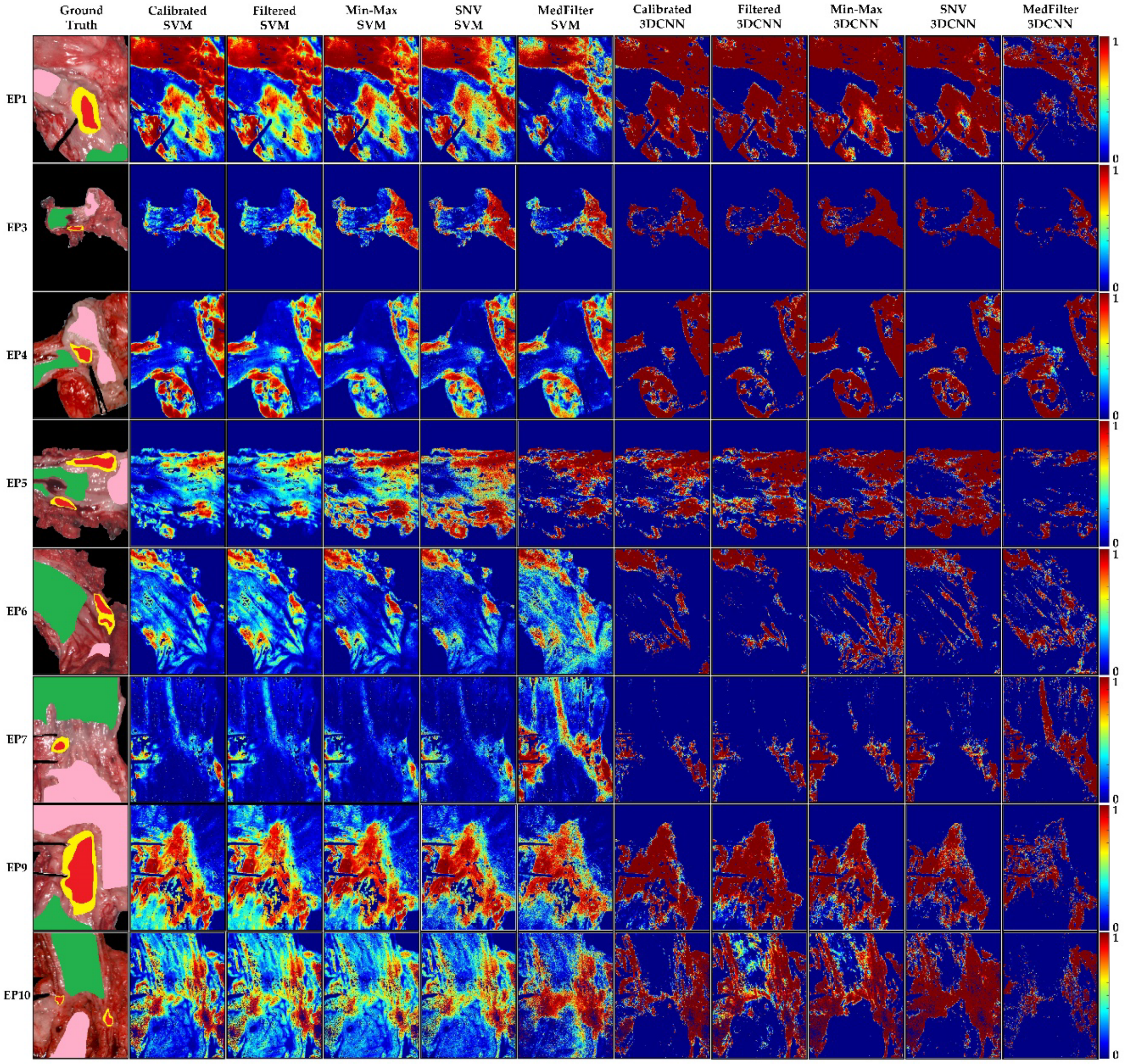

Figure S4. Tumor class probability maps results of the esophagogastric database in each patient.

Evaluation of Preprocessing Methods on Independent Medical Hyperspectral Databases to Improve Analysis

(Supplementary Material)

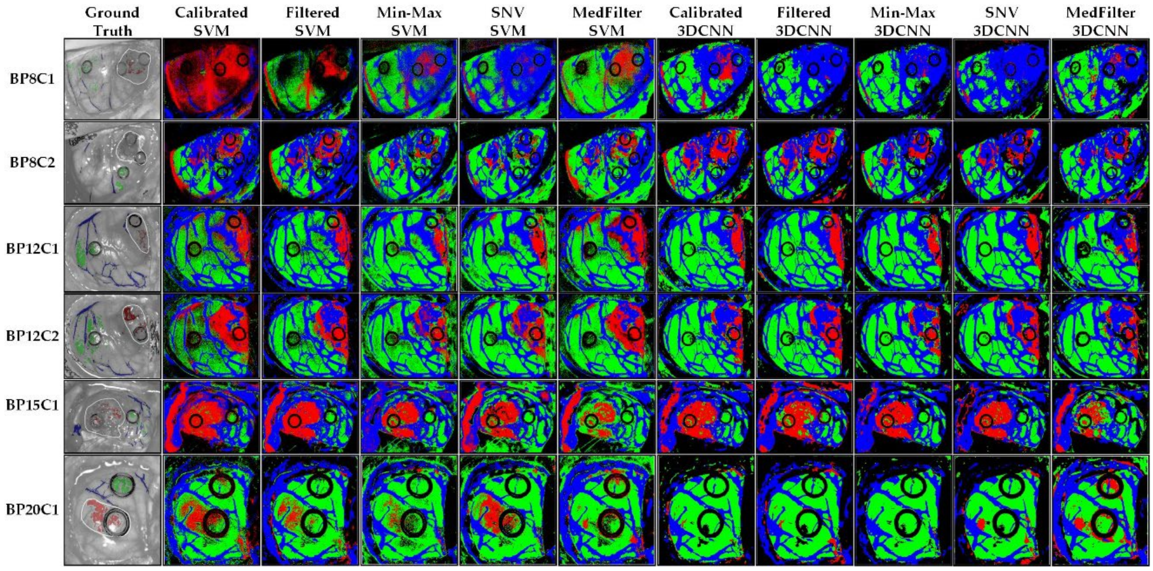

Figure S5. Classification maps results of each model of the brain database each patient. Green color indicates brain healthy tissue, red color represents tumor tissue, blue color identifies the blood vessels and black represent the background.

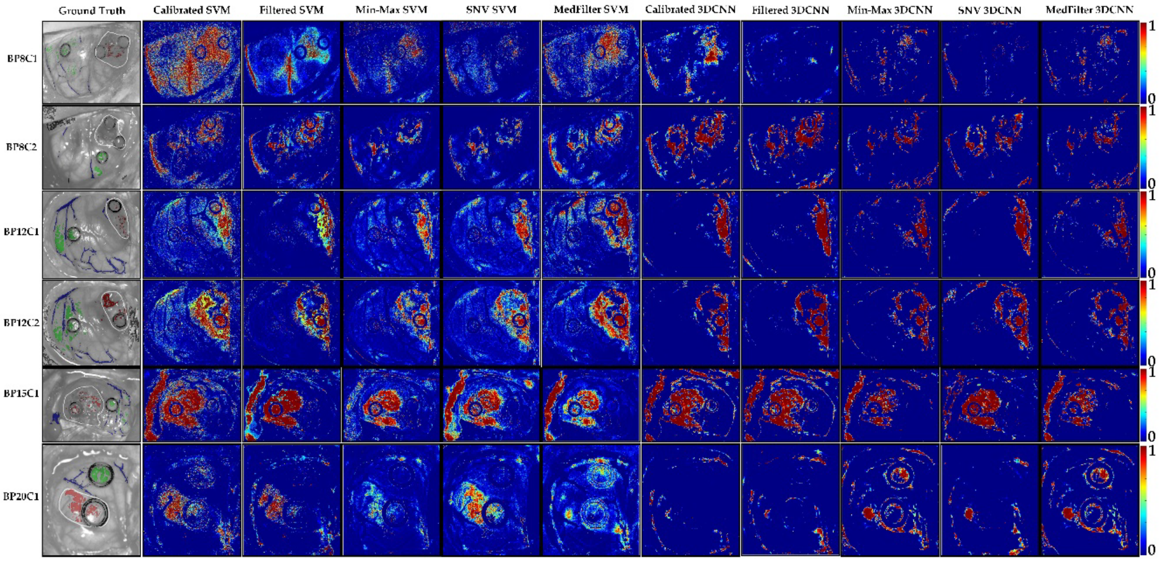

Figure S6. Tumor class probability maps results of the brain database in each patient.
